# Chemotherapy-Induced Atrial Fibrillation With Rapid Ventricular Response in a Patient With Pleomorphic Rhabdomyosarcoma: A Case Report and Approach to Management

**DOI:** 10.7759/cureus.39375

**Published:** 2023-05-23

**Authors:** Mubariz A Hassan, Yashvardhan Batta, Muhammad Adil Afzal, Niyati Grewal

**Affiliations:** 1 Internal Medicine, Howard University Hospital, Washington, D.C., USA; 2 Internal Medicine, St. Joseph's Regional Medical Center, Paterson, USA

**Keywords:** atrial fibrillation, chemotherapy-associated cardiotoxicity, af rate control, atrial fibrillation management, pleomorphic rhabdomyosarcoma, atrial fibrillation (af)

## Abstract

Atrial fibrillation (AF) is the most common sustained cardiac arrhythmia. Its prevalence in cancer patients undergoing treatment with radiation or chemotherapeutic agents has been on the rise. The most common offending agents are alkylating agents and anthracyclines causing various types of arrhythmias, including AF. We report a case of a 62-year-old male who was diagnosed with stage IV pleomorphic rhabdomyosarcoma and was started on chemotherapy with a mesna-ifosfamide and doxorubicin (MAI) regimen. He developed AF with a rapid ventricular rate soon after his second cycle of treatment, which got better with the initiation of beta-blocker therapy. Since low blood counts, including low platelet levels, are expected in patients with chemotherapy, the continual use of anticoagulation therapy varies on a case-to-case basis.

## Introduction

Atrial fibrillation (AF) is one of the most common cardiac arrhythmias in the general population along with atrial flutter [1]. It is estimated that three to six million people have been diagnosed with AF in the United States alone [2]. The most common risk factors for AF are hypertension, diabetes, advancing age, rheumatic and non-rheumatic valve disease, congestive heart failure, myocardial infarction, and certain drugs. Although cardiotoxicity in the form of cardiomyopathy has been well-reported by chemotherapeutic agents, chemotherapy-induced arrhythmias are also becoming more common [3]. It is surprising to see how quickly these arrhythmias, especially AF, develop in cancer patients after starting chemotherapy, as seen in our patient. A wide range of chemotherapy agents have been associated with cardiotoxicity for which the anthracyclines and related compounds like doxorubicin and daunorubicin are more frequently implicated. Most of the cardiotoxicity by these agents involves the development of heart failure or any kind of arrhythmias, in which the time to presentation can vary from weeks to months of exposure. Arrhythmias associated with chemotherapy are usually treated with cessation of the causative agent along with acute and chronic management of the arrhythmias.

## Case presentation

**Figure 1:**
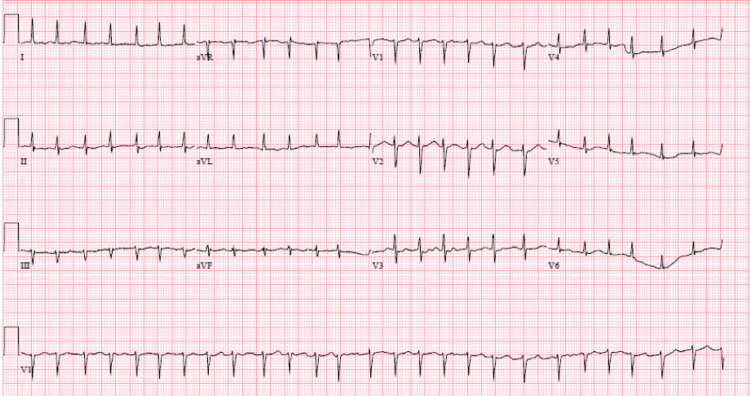
Atrial fibrillation with rapid ventricular response

The patient was a 62-year-old man with a past medical history significant for transverse myelitis (on CellCept), deep vein thrombosis (on therapeutic anticoagulation with Eliquis), and hypertension (well controlled with oral antihypertensive), who presented initially to the outpatient care setting with a complaint of left-sided hip pain for two months. He assumed it was arthritis pain and did some physical therapy exercises with over-the-counter analgesics but it did not improve. His symptoms got worse and according to the patient, he started to feel a knot-like sensation in his left hip that prompted further investigation and workup with imaging. Magnetic resonance imaging (MRI) showed a large soft tissue mass in the proximal left thigh with abnormal bone marrow signals with a skip lesion. A biopsy was done that confirmed the diagnosis of high-grade pleomorphic rhabdomyosarcoma. Oncology was consulted and a plan was made to start the treatment with radiation and chemotherapy. On exam, the patient was not in any acute distress with a temperature of 98.7°F, heart rate of 85 beats per minute, respiratory rate of 14 breaths per minute, and blood pressure of 121/78 mmHg, along with oxygen saturation of 99% on room air. Basic lab work was done that was unremarkable for any electrolyte abnormalities and thyroid function tests were not consistent with hyperthyroidism. Baseline ECG and cardiac echocardiogram were done that showed no structural abnormalities of the heart with normal ejection fraction. The patient started chemotherapy with mesna-ifosfamide and doxorubicin (MAI) regime, including a combination of anthracycline and alkylating agents. The patient tolerated the first session of therapy fairly well and presented for his second cycle three weeks later with no pertinent complaints. Soon after he was given the second session of his chemotherapy, the patient developed cardiac arrhythmias, with noticeable atrial fibrillation with a sustained rapid ventricular rate of up to 160-180 beats per minute (Figure 1). Cardiology was consulted and a shared decision was made in terms of rate vs. rhythm control strategy. The patient was started on beta blockers along with digoxin that controlled his heart rate and was discharged home with cardiology and oncology follow-up.

## Discussion

Chemotherapy is a cornerstone of cancer therapeutic management, but it is not without potential complications. Although the field of cancer therapeutics has undergone dramatic changes over subsequent decades, anthracyclines remain the cornerstone of contemporary chemotherapeutic regimens for a variety of cancers. Among the most significant concerns is the risk of cardiotoxicity associated with the use of anthracyclines, which exert their cytotoxic effects via inducing DNA damage, inflammation, oxidative stress, and mitochondrial dysfunction in cardiomyocytes [4]. Although the mechanisms underlying anthracycline-induced cardiotoxicity are not fully elucidated, several pathways have been implicated, including reactive oxygen species, lipid peroxidation, iron-mediated damage, and disruption of calcium homeostasis. The main mechanism of anthracycline cardiotoxicity is now thought to be through the inhibition of topoisomerase 2 beta, resulting in the activation of the cell death pathway as well as the inhibition of mitochondrial biogenesis.

Anthracycline-induced cardiotoxicity is cumulative, dose-dependent, and augmented by risk factors such as age, pre-existing cardiovascular disease, and concomitant administration of drugs with potential cardiovascular effects. Doxorubicin, a well-known anthracycline, is associated with ventricular dysfunction and dilated cardiomyopathy, increasing risks for arrhythmias such as AF, which can be risk factors for the development of arrhythmias, such as AF with an incidence rate of up to 15% [5]. Sinus tachycardia, supraventricular tachycardia, and ventricular arrhythmias may also be implicated [6].

Preventing anthracycline-induced cardiotoxicity requires a multidisciplinary approach involving pharmacologists, hematologists, oncologists, cardiologists, and primary care providers. Strategies to reduce the risk of cardiotoxicity include reducing cumulative dosing, monitoring cardiac function with serial echocardiographic studies, and managing patient-specific cardiovascular risk factors such as hypertension and dyslipidemia with appropriate medical interventions. Furthermore, cardioprotective chelating agents, such as dexrazoxane, have been shown to reduce free radical damage, and beta-blockers, angiotensin-converting enzyme inhibitors, and angiotensin receptor blockers have been found to be effective in preventing arrhythmias in patients undergoing doxorubicin chemotherapy [7,8].

To manage patients effectively and prevent complications, a shared decision-making approach between teams is critical. This includes discussions on rate versus rhythm control strategies, the selection of appropriate pharmacological interventions, and careful monitoring of patient-specific cardiovascular risk factors. Further research is necessary to better understand the underlying mechanisms of anthracycline-induced cardiotoxicity, identify high-risk patients, implement preventative measures, and improve outcomes.

## Conclusions

In conclusion, anthracycline cardiotoxicity remains a significant clinical concern in cardiology and oncology. As the survival of cancer patients continues to improve, long-term cardiovascular complications of chemotherapy are a major concern. Clinicians must continue to recognize the importance of multidisciplinary care, adopt appropriate monitoring strategies, and use preventative measures to avoid potential chemotherapy complications. The decision to address rate vs. rhythm control for AF varies from case to case and requires a holistic approach in terms of medical management along with the use of anticoagulation concomitant with chemotherapy. A goal in the emerging field of cardio-oncology is to make sure cancer treatments are safer from a cardiovascular perspective. Enhancing our comprehension of the mechanisms underlying cardiotoxicity can facilitate the development of improved strategies for monitoring, prevention, and management.
